# Comparative Analysis of Multi-Omics Integration Using Graph Neural Networks for Cancer Classification

**DOI:** 10.1109/access.2025.3540769

**Published:** 2025-02-11

**Authors:** FADI ALHARBI, ALEKSANDAR VAKANSKI, BOYU ZHANG, MURTADA K. ELBASHIR, MOHANAD MOHAMMED

**Affiliations:** 1 College of Engineering, Department of Computer Science, University of Idaho, Moscow, ID 83844, USA; 2 College of Computer and Information Sciences, Department of Information Systems, Jouf University, Sakaka, Al-Jouf 72441, Saudi Arabia; 3 School of Mathematics, Statistics and Computer Science, University of KwaZulu-Natal, Pietermaritzburg 3209, South Africa

**Keywords:** Cancer classification, gene expression analysis, graph neural networks, multi-omics data integration, correlation matrices, protein-protein interaction networks

## Abstract

Recent studies on integrating multiple omics data highlighted the potential to advance our understanding of the cancer disease process. Computational models based on graph neural networks and attention-based architectures have demonstrated promising results for cancer classification due to their ability to model complex relationships among biological entities. However, challenges related to addressing the high dimensionality and complexity in integrating multi-omics data, as well as in constructing graph structures that effectively capture the interactions between nodes, remain active areas of research. This study evaluates graph neural network architectures for multi-omics (MO) data integration based on graph-convolutional networks (GCN), graph-attention networks (GAT), and graph-transformer networks (GTN). Differential gene expression and LASSO (Least Absolute Shrinkage and Selection Operator) regression are employed for reducing the omics data dimensionality and feature selection; hence, the developed models are referred to as LASSO-MOGCN, LASSO-MOGAT, and LASSO-MOGTN. Graph structures constructed using sample correlation matrices and protein-protein interaction networks are investigated. Experimental validation is performed with a dataset of 8,464 samples from 31 cancer types and normal tissue, comprising messenger-RNA, micro-RNA, and DNA methylation data. The results show that the models integrating multi-omics data outperformed the models trained on single omics data, where LASSO-MOGAT achieved the best overall performance, with an accuracy of 95.9%. The findings also suggest that correlation-based graph structures enhance the models’ ability to identify shared cancer-specific signatures across patients in comparison to protein-protein interaction networks-based graph structures. The code and data used in this study are available in the link (https://github.com/FadiAlharbi2024/Graph_Based_Architecture.git).

## I. INTRODUCTION

Gene expression is a fundamental cellular process that governs the translation of genetic information encoded in DNA into functional proteins, which in turn define a cell’s functionality and phenotype [1]. Recent advancements in gene expression analysis have significantly enhanced our understanding of the molecular pathology of various diseases, including cancer [2]. Particularly, the advent of high-throughput sequencing technologies has enabled genome-wide analysis of gene expression patterns, providing critical insights into tumorigenesis and cancer rogression [3]. Moreover, the integration of gene expression data with other molecular datasets has proven effective for biomarker characterization, facilitating early cancer diagnostics and treatment methods selection [4]. Incorporating multiple layers of omics data enables identification of distinct molecular subtypes that might remain undetected with single-omics analyses.

The analysis and interpretation of gene expression data have been facilitated by Machine Learning (ML)-based methods, due to the ability for pattern recognition in complex high-dimensional data. Consequently, ML models have been successfully applied to predict disease progression, differentiate cancer subtypes, and identify therapeutic targets with high levels of accuracy [5]. For instance, ML methods based on Random Forest, Support Vector Machines, and Neural Networks have been employed as feature selection tools to identify the most relevant genes within gene expression data for specific biological processes [6], [7], [8]. In addition, Deep Learning (DL) models have been essential in integrating multi-omics data by uncovering patterns and relationships within large datasets [9]. DL-based approaches, in particular, have been extensively used to integrate gene expression data with other types of omics data, such as proteomics and metabolomics, to improve cancer prognosis through reductionist modeling [10].

Graph Neural Networks (GNNs) have recently emerged as powerful tools for analyzing data with relational structures, such as biological networks, social networks, and knowledge graphs. GNNs are designed to operate on data represented in the form of a graph, where nodes and edges model the relationships within the graph elements [11]. In the context of biological networks, GNNs have proven effective in capturing complex interactions between biological entities, e.g., in the form of protein-protein interaction networks and gene regulatory networks [12]. By representing these interactions as graphs, GNNs model the dependencies and hierarchical structures within biological systems, providing important insights into cellular processes. Recent emerging graph-based network architecture, such as Graph Convolutional Networks (GCNs), Graph Attention Networks (GATs), and Graph Transformer networks (GTNs), have further enhanced the performance of GNNs, enabling more accurate and interpretable outcomes [13]. GCNs extend the concept of convolution from traditional grid-based data, such as images, to graph structures, enabling the capture of information from a node’s neighbors and creating a localized graph representation around a node [14]. This makes GCNs especially effective for tasks such as node classification and link prediction, where relationships between neighboring nodes are particularly important. GATs build on this concept by incorporating an attention mechanism, allowing the model to assign different weights to neighboring nodes for learning from complex graphs [15]. The attention mechanism enables GATs to concentrate on the important parts of the graph, improving performance on tasks involving heterogeneous graphs or graphs where certain connections are more significant. GTNs introduce transformer network architectures into graph learning, providing the ability to handle long-range geometric dependencies within the graph [16]. GTNs are particularly helpful for graph-level prediction tasks, as they focus on learning global features across the entire graph. Together, GCNs, GATs, and GTNs graph-based architectures contribute to advanced modeling of complex data structures enabling more accurate solutions across a range of tasks.

This study provides an empirical evaluation of the performance of GCNs, GATs, and GTNs for classifying 31 types of cancers and normal tissues. The focus is on evaluating the capacity for integrating multi-omics data, including different combinations of mRNA (messenger-RNA), miRNA (micro-RNA), and DNA methylation data. Our approach also applies LASSO (Least Absolute Shrinkage and Selection) regression for feature selection. Therefore, the compared approaches are referred to as LASSO-MOGCN (Multi-Omics Graph Convolutional Network), LASSO-MOGAT (Multi-Omics Graph Attention Network), and LASSO-MOGTN (Multi-Omics Graph Transformer Network). For modeling the relationships between the variables in the graph-based architectures we used patients’ correlation matrices and protein-protein interaction (PPI) networks to capture biological interaction and interdependence. The experimental validation reveals a notable difference in the performance between single-omics data and multi-omics data, where the use of single omics data generally resulted in lower performance compared to the integration of more than one omics data type. For example, LASSO-MOGAT achieved an accuracy of 94.88% when with DNA methylation alone as input, 95.67% accuracy for integrating mRNA and DNA methylation, and 95.90% accuracy based on integration of mRNA, miRNA, and DNA methylation data. The improvement in the model performance highlights the importance of multi-omics integration by leveraging complementary information from different data types. Among the three approaches, LASSO-MOGAT provided the best performance for both single-omics and multi-omics data.

Unlike previous related works, this study compares three graph-based models using multi-omics data (mRNA, miRNA, DNA methylation) to classify 31 different types of cancer and normal tissues. While graph-based methods have recently been widely applied for cancer classification [17], [18], [19], [20], [21], few studies have focused on systematically comparing modern graph-based architectures and their performances across diverse omics data. For instance, Ramirez et al. [22] employed GCNs for cancer classification using co-expressed genes and PPI networks. Nevertheless, their study did not investigate the performance of other graph architectures, such as GATs or GTNs. Schulte-Sasse et al. [23] introduced Explainable Multi-Omics Graph Integration (EMOGI), primarily targeting cancer gene prediction, but their work did not compare different GNN architectures for multi-omics integration. Different from prior works, our study presents a systematic comparison of GCNs, GATs, and GTNs for omics integration based on different graph structures. Furthermore, unlike existing studies, we focus on feature selection with LASSO regression while using graph-based techniques for handling complex associations between multiple omics datasets. In a previous work by our team, we introduced a multi-omics GAT framework [24], which utilizes the PPI network as a graph structure. While our previous work focused on developing a GAT model for multi-omics analysis, in the work presented in this paper, we evaluate three graph models, GCNs, GATs, and GTNs, based on both correlation matrices and PPI networks as graph structures.

The key contributions of this study are as follows:

- An empirical investigation of the performance of GCNs, GATs, and GTNs based on integrating mRNA, miRNA, and DNA methylation data for classification of 31 types of cancer and normal tissues.
- A comparative analysis between the performance of two graph structures based on correlation matrices and PPI networks.
- The performance of LASSO-MOGAT utilizing correlation matrices demonstrated state-of-the-art performance in comparison to existing graph-based models using multi-omics data integration for cancer classification.

### A. RELATED WORK

In recent years, studies integrating multiple omics data sets have exhibited potential to advance cancer research by providing an improved understanding of the disease process. Computational models based on deep learning and attention-based architectures were applied to classify subtype cancer, enhance model interpretability, and identify biomarkers. However, challenges related to models’ applicability for other omics data, model interpretability, and clinical applicability, as well as challenges with regard to analyzing multiple omics data with high dimensions persist.

Recently, Xiao et al. [25] introduced Multi-Prior Knowledge Graph Neural Network (MPKGNN) for cancer molecular subtype classification. The authors demonstrated that the incorporation of multiple Prior Knowledge Graphs in GNNs increases the volume of omics data that it is capable of classifying with high accuracy. Chatzianastasis et al. [26] developed Explainable Multilayer Graph Neural Network (EMGNN) for cancer gene prediction using gene interaction graphs and multi-omics data. This work identifies more than one interaction network for improved performance, however the ability for generalization to high dimensions and large-scale datasets remains unexplored.

GCNs have also been used for cancer data analysis by integrating patient similarity networks with other omics data. For instance, Moon and Lee [27] developed the Multi-Omics Module Analysis (MOMA) model, and Li et al. [28] introduced the Multi-omics Graph Convolutional Network (MoGCN). However, these works do not account for the various aspects of multiple omics data integration, especially the inter-omics associations essential in the classification of subtypes of cancer. Specifically, in MoGCN by Li et al. [28] GCNs are combined with autoencoders for feature learning, but Similarity Network Fusion formulates a significant portion of interactions across multiple omics layers which are important and can be neglected.

Wang et al. [29] proposed Multi-Omics Graph Convolutional Networks (MOGONET), which includes a cross-omics correlation learning process. Although this study is more exhaustive, it also has drawbacks regarding the model interpretability, as it is unclear how individual biomarkers are chosen for a particular disease. Likewise, Schulte-Sasse et al. [23] and Peng et al. [30] target enhancing biomarker discovery, however, the inability to clearly explain the model decision-making hampers their utility. This challenge has been mitigated by the use of attention mechanisms. Zhang et al. [31] and Guo et al. [32] applied attention-based GCNs for cancer classification with improved model explanations of cancer classification and patient biomarkers. Nevertheless, these models pose challenges in terms of scalability, generalizability, and robustness for complex multi-omics datasets.

GTNs are also promising architectures as they are capable of capturing long-range dependencies in complex biological data. Kaczmarek et al. [33] and Wang et al. [34] applied GTNs for cancer classification, combining multi-omics data with miRNA and mRNA sequencing data. Although these models offer better interpretability, especially in the identification of key biological pathways, they perform poorly in terms of accuracy compared to the traditional deep learning methods, thus a better combination of omics fusion techniques is required. There have also been works on application of autoencoders for dimensionality reduction in multi-omics data. Although the works by Zhang et al. [13] and Chai et al. [35] achieved high cancer classification accuracy, autoencoders are generally considered a black-box solution that lacks explanation, which can critically impact the utility of these algorithms. Additionally, due to the lack of ability to decode the relations of different omics, autoencoders have shortcomings in comparison to feature learning with graph-based methods.

The study by Khemani et al. [36] explored certain GNN models, such as GATs, GCNs, and GraphSAGE that are currently widely employed in many different applications. Similarly, Lachi et al. [37] investigated scalable multi-graph pretraining. These studies addressed some of the prevalent challenges for generalizability and performance gain in graph learning. Although these works offer a comparison of advanced computational models, they fail to answer questions about the optimal level of balance between performance and interpretability in multi-omics data analysis. Besides, research works aiming at solving problems on a larger scale, such as Zhao et al. [38] and Yang et al. [39], struggle with algorithmic issues of GNN models to process large and complex multi-omics data sets.

In conclusion, while efforts using graph-based architecture for cancer classification and biomarker identification have yielded promising results, several limitations remain. Prior approaches tend to prioritize performance or interpretability, nonetheless, models that effectively balance both characteristics are relatively scarce, particularly in the context of large, heterogeneous multi-omics data integration. Furthermore, the integration of omics data types including RNA-Seq, miRNA, and DNA methylation is still challenging because most approaches do not capture the intricacies of the relationships among the data types. Consequently, developing approaches that are capable of unifying multi-omics data in interpretable and computationally efficient frameworks, and with a high prediction power remains an open research question.

## II. MATERIALS AND METHODS

### A. DATA COLLECTION

**FIGURE 1.**
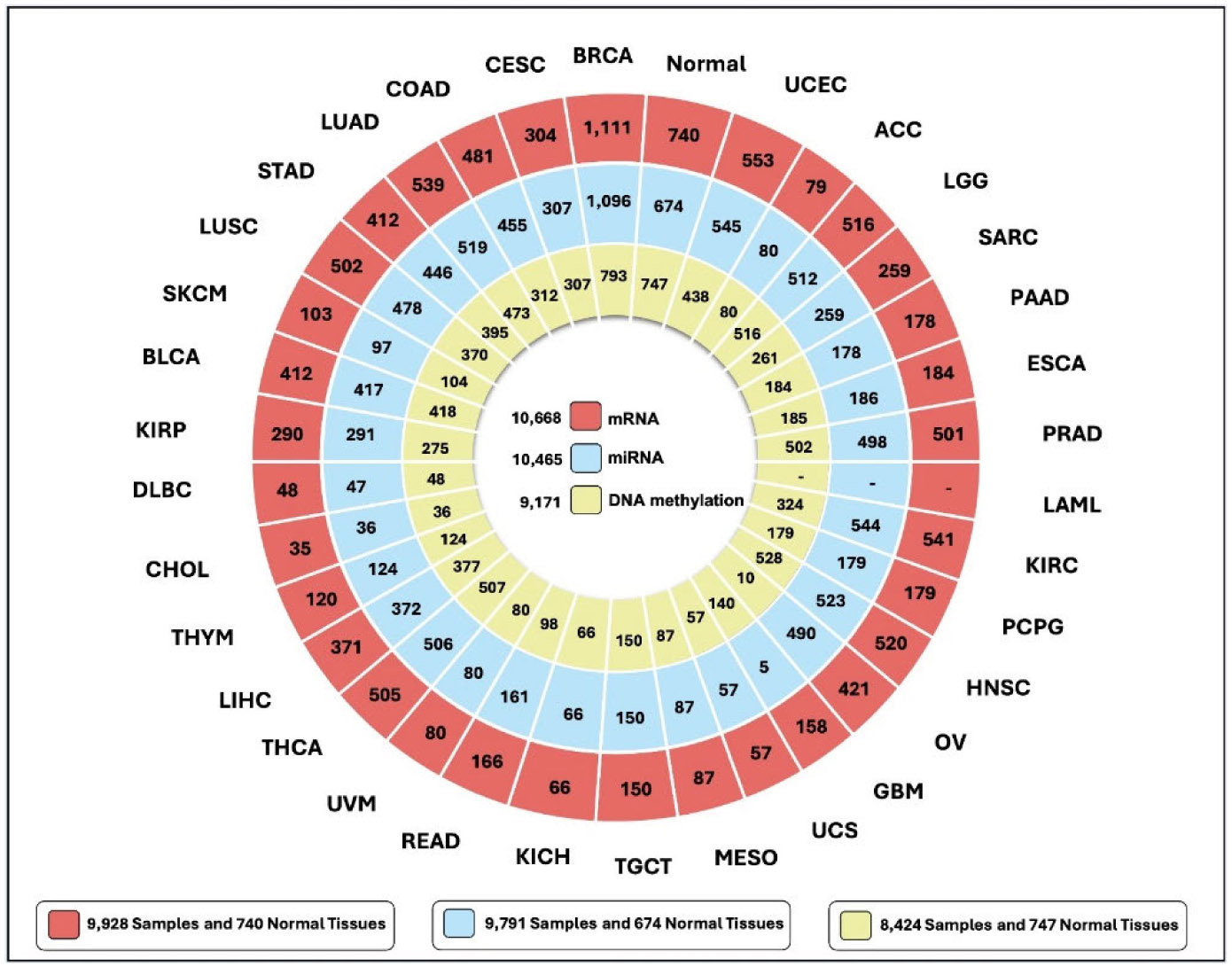
Tumor types including the number of samples and normal tissues of TCGA Multi-omics data (mRNA, miRNA, and DNA methylation) used in this study.

The multi-omics data for the different cancers used in this study were obtained from the Pan-Cancer Atlas [40] using the GDC query tool from the TCGAbiolinks library [41]. The Genomic Data Commons is a project set up by the National Cancer Institute which is dedicated to providing a central database that can be used by researchers wishing to carry out cancer genomic studies. The tumor types and the number of samples in the multi-omics data (mRNA, miRNA, and DNA methylation) used in this study are depicted in Figure 1. In total, the initial dataset consisted of 10,668 samples with mRNA data, 10,465 samples with miRNA data, and 9,171 samples with DNA methylation data.

### B. DATA PREPROCESSING

The pipeline for data preprocessing in this study includes techniques for identifying relevant biological features within omics data and techniques for dimensionality reduction to select informative features in high-dimensional data. To identify the relevant biological features, we used differential gene expression (DGE) analysis and LIMMA. In particular, DGE analysis was performed with DESeq2, which employs a negative binomial model to identify genes from mRNA data that have significant changes in the gene expression levels [41], [42]. LIMMA was applied to DNA methylation data to identify significant differentially methylated CpG sites [43]. Afterward, LASSO regression was applied to extend the features selection process for mRNA and DNA methylation data and reduce the dimensionality of the omics data [44]. The steps in the data preprocessing pipeline are described in the next sections and the entire feature selection process is summarized in Table 1.

#### 1) DIFFERENTIAL GENE EXPRESSION (DGE) ANALYSIS

DGE profiling is widely used in genomics to compare the levels of gene expression in a given organism under different conditions or environments (for example, treatment versus control, or normal versus cancer, etc.) [45]. This helps understanding how genes are regulated and their actions depending on the environmental conditions and a host of other functions. For the current study, we performed differential gene expression on the mRNA data using the DESeq2 package. This method takes a general linear model of the data count for every gene using negative binomial distribution to capture both biological variation and overdispersion. The values of the estimated log fold changes were tested for their significance by the Wald test based on the *p*-values obtained from the Wald statistic. To identify genes that are likely to be significant for the studied biological processes, we set the p-value threshold to 0.001.

#### 2) LIMMA

We applied the LIMMA (Linear Models for Microarray) model to perform differential methylation analysis by fitting a linear model for the methylation levels of CpG sites as a function of the experimental sample groups [46]. The dataset has 9,171 samples and 485,577 features obtained from the Human Methylation 450K (HM450) array [47]. Using the LIMMA model, we selected CpG sites significantly methylated in tumors compared to normal samples. LIMMA provides a moderated t-statistic for each CpG site and provides an estimate of the effect size that reflects the relative difference in methylation between the groups. The p-value that corresponds to the t-statistic indicates the statistical significance of the differences. We used a cutoff p-value of 0.05 to filter the data, and thus the number of features was reduced to 139,321 features that concentrate on the most significant methylation changes.

#### 3) LASSO

Lasso regression is a linear regression method with an additional regularization term in the objective function, defined as:

(1) $$\underset{\beta }{\text{min}}\left\{\frac{1}{2n}\sum_{i=1}^{n}{\left({𝒴}_{i}-X_{i}\beta \right)}^{2}+\lambda \sum_{j=1}^{p}|\beta_{j}|\right\}$$

The first term in (1) is the residual sum of squared differences, and the second term is $L_{1}$ penalty that encourages the model to learn sparse coefficients. Adjusted by the parameter λ, Lasso regression establishes a balance between the fit and the complexity of the model. LASSO enables selecting a final set of the most significant features for further analysis.

### C. MULTI-OMICS DATA INTEGRATION

**FIGURE 2.**
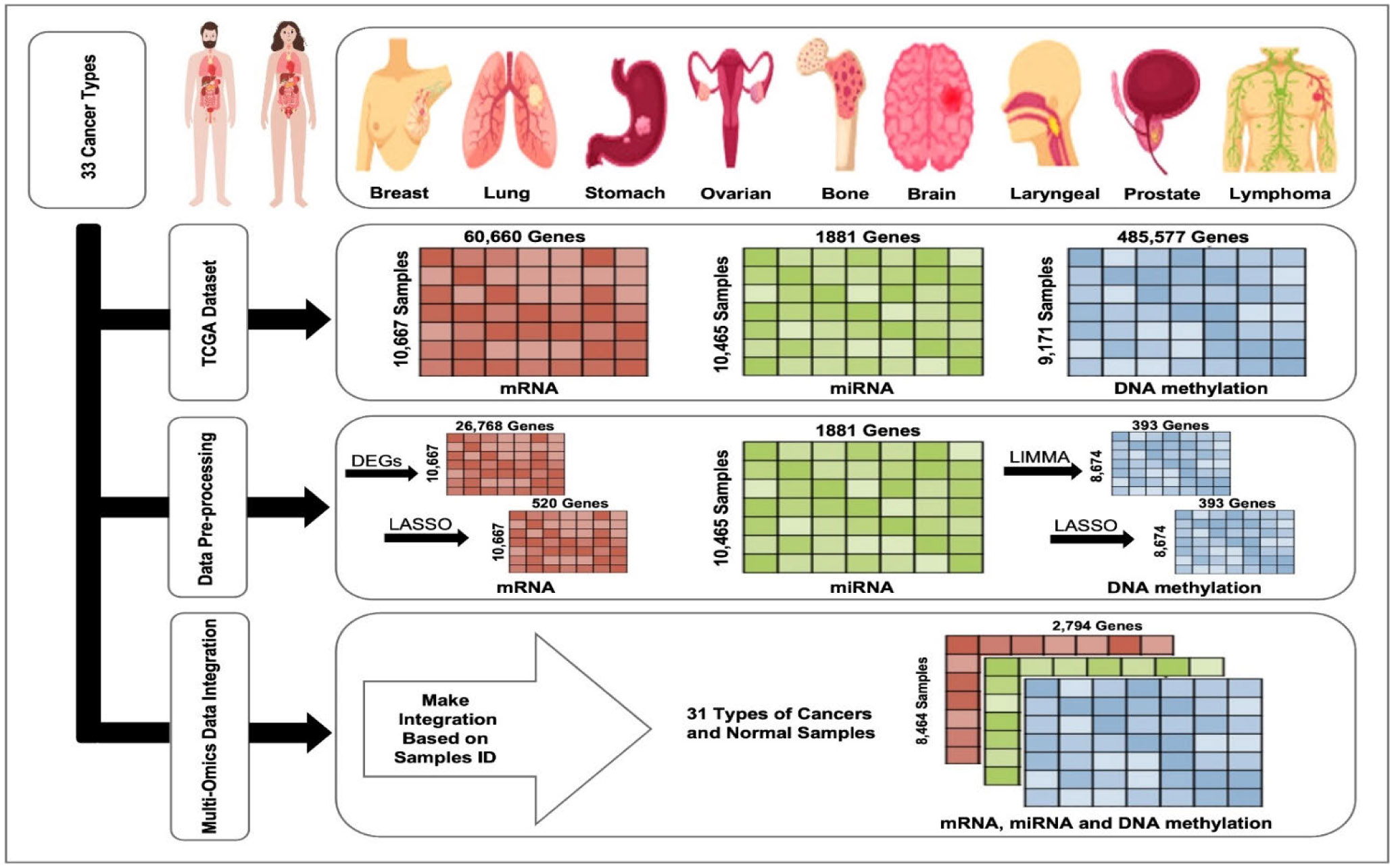
Preprocessing steps and data integration. Omics data (mRNA, miRNA, and DNA methylation) were obtained from the Pan-cancer Atlas using the TCGAbiolinks library. Next, differential expression analysis (DEG) and LASSO regression were applied to mRNA data, while LIMMA and LASSO regression were applied to DNA methylation data. Subsequently, mRNA or RNA-Seq, miRNA, and DNA methylation data.

We merged all omics data pertaining to each sample into a single record, integrating mRNA or RNA-Seq, miRNA, and DNA methylation data using the sample ID as a common value. The inner join merging operation was used on the sample ID of the three datasets and only samples with the complete data in all the three omics data were retained. Cancer types for which omics data was incomplete (for instance, ‘TCGA_LAML’ cancer type have no RNA-Seq data, and ‘TCGA_GBM’ have no miRNA data) were omitted from further analysis. The final dataset comprises 8,464 samples involving 2,794 omics features and covers 31 cancer types and normal tissues. The preprocessing steps and data integration are illustrated in Figure 2.

### D. GRAPH STRUCTURES

#### 1) CORRELATION MATRICES

For the graph construction, we employed two types of graph structures: sample correlation matrices and the molecular topology of PPI networks. Sample correlation matrices capture relationships by constructing edges based on the similarity between samples to define the graph structure for graph-based learning methods. Prior works reported that samples from the same cancer type tend to have more similar omics profiles [48]. Therefore, two samples from the same cancer type are likely to show a strong correlation in their profiles [49]. Previous studies have also leveraged sample-sample correlation matrices effectively for the accurate classification of biomedical cases [29]. In this study, the correlation matrix for each omics data type was computed using the Pearson correlation coefficient to quantify the relationship between pairs of features in defining the graph structure. The final correlation matrix was obtained by setting a threshold at 0.9. The correlation coefficient between pairs of samples is calculated as follows:

(2) $$\mathrm{corr}\left(S_{i},S_{j}\right)=\frac{\mathrm{cov}(S_{i},S_{j})}{\sigma_{S_{i}}\sigma_{S_{j}}}$$

where $\mathrm{corr}\left(S_{i},S_{j}\right)$ denotes the covariance between $S_{i}$ and $S_{j}$, $\sigma_{S_{i}}$ is the standard deviation of $S_{i}$, and $\sigma_{S_{j}}$ is the standard deviation of $S_{j}$.

#### 2) PROTEIN-PROTEIN INTERACTION (PPI) NETWORK

The PPI network depicts the relationship existing between proteins in a cell [50], [51]. Proteins interact to conduct numerous biological processes including metabolism, gene regulation, and signaling, and therefore PPI networks are crucial for describing pathophysiological processes like cancer and other cellular processes [52], [53]. The PPI network was constructed based on the corresponding genes from the STRING database [54]. In PPI networks, the nodes refer to proteins and edges refer to the known relationships between them [50]. This structure involves the identification of the proteins as well as the strength of the interaction between them. The nodes and edges were coded as tensors, and afterward were fed into the graph network architectures. In this graph-based representation, each sample is described by the topology of the PPI network incorporating nodes with multi-omics features obtained from DNA methylation, miRNA, and mRNA.

### E. GRAPH NEURAL NETWORK ARCHITECTURES

#### 1) GRAPH CONVOLUTIONAL NETWORKS (GCNs)

GCNs generalize the concept of convolution common for traditional grid data, such as images, to graph data. In GCNs, a node aggregates information from its neighboring nodes to compute its features, and thus the model captures the localized spatial structure of a graph. The neighborhood aggregation mechanism effectively consolidates information from connected nodes, capturing complex dependencies that would be missed by non-graph-based models [22]. For instance, GCNs may model the protein-protein relations or merge various types of omics data for cancer typing. The layer-wise propagation rule for the GCNs using the correlation matrix or PPI network graph structure can be defined as [12]:

(3) $$H^{\left(l+1\right)}=\sigma (D^{-\frac{1}{2}}AD^{-\frac{1}{2}}H^{\left(l\right)}W^{\left(l\right)})$$

$H^{\left(l\right)}$ stands for the feature matrix at layer 1, $W^{\left(l\right)}$ is a weight matrix, A is an adjacency matrix, D is the degree matrix, and σ is an activation function [14].

#### 2) GRAPH ATTENTION NETWORKS (GATs)

GATs are neural network architectures designed for learning data representations in graphs by applying an attention mechanism to the nodes of the graph. The goal of GATs is to develop weighting factors that consider both the importance of node attributes and node degrees. The nodes’ representations are updated by combining information from neighboring nodes. While GATs utilize an attention-based neighborhood aggregation mechanism to distribute scaling factors, GCNs use a non-parametric scaling factor that is obtained from a normalizing function [55]. During neighborhood aggregation, GATs can assign larger weights to more significant nodes.

The attention mechanism for the feature update rule of a node is given as [56]:

(4) $$h_{i}=\sigma (\sum_{j\in 𝒩(i)}\alpha_{ij}Wh_{j})$$

where $h_{i}$ is the updated feature of node i, σ is an activation function, 𝒩(i) is the set of neighbors of node i, $\alpha_{ij}$ is the attention coefficient between node i and its neighbor j, which measures the importance of node j’s features to node i. W is a learnable weight matrix applied to the features.

The attention coefficients $\alpha_{ij}$ are computed using [15]:

(5) $$\alpha_{ij}=\frac{\text{exp}(\text{LeakyReLU}(a^{T}[Wh_{i}\|Wh_{j}]))}{\sum_{k\in 𝒩(i)}\text{exp}(\text{LeakyReLU}(a^{T}[Wh_{i}\|Wh_{k}]))}$$

where $a^{T}$ is a learnable attention vector.

#### 3) GRAPH TRANSFORMER NETWORKS (GTNs)

GTNs utilize the architecture of Transformer networks, allowing to learn long-range dependencies, as well as embedded interactions inside the graph. GTNs use a global attention mechanism that allows each node to attend to any node in the entire graph. This approach covers both shortrange and long-range dependencies, which makes it useful in tasks where finer details of the structure of the graph are very important. Graph transformers update nodes using self-attention, which recognizes the information that other nodes contain through attention scores before combining the specific features with weighted information from the graph.

**FIGURE 3.**
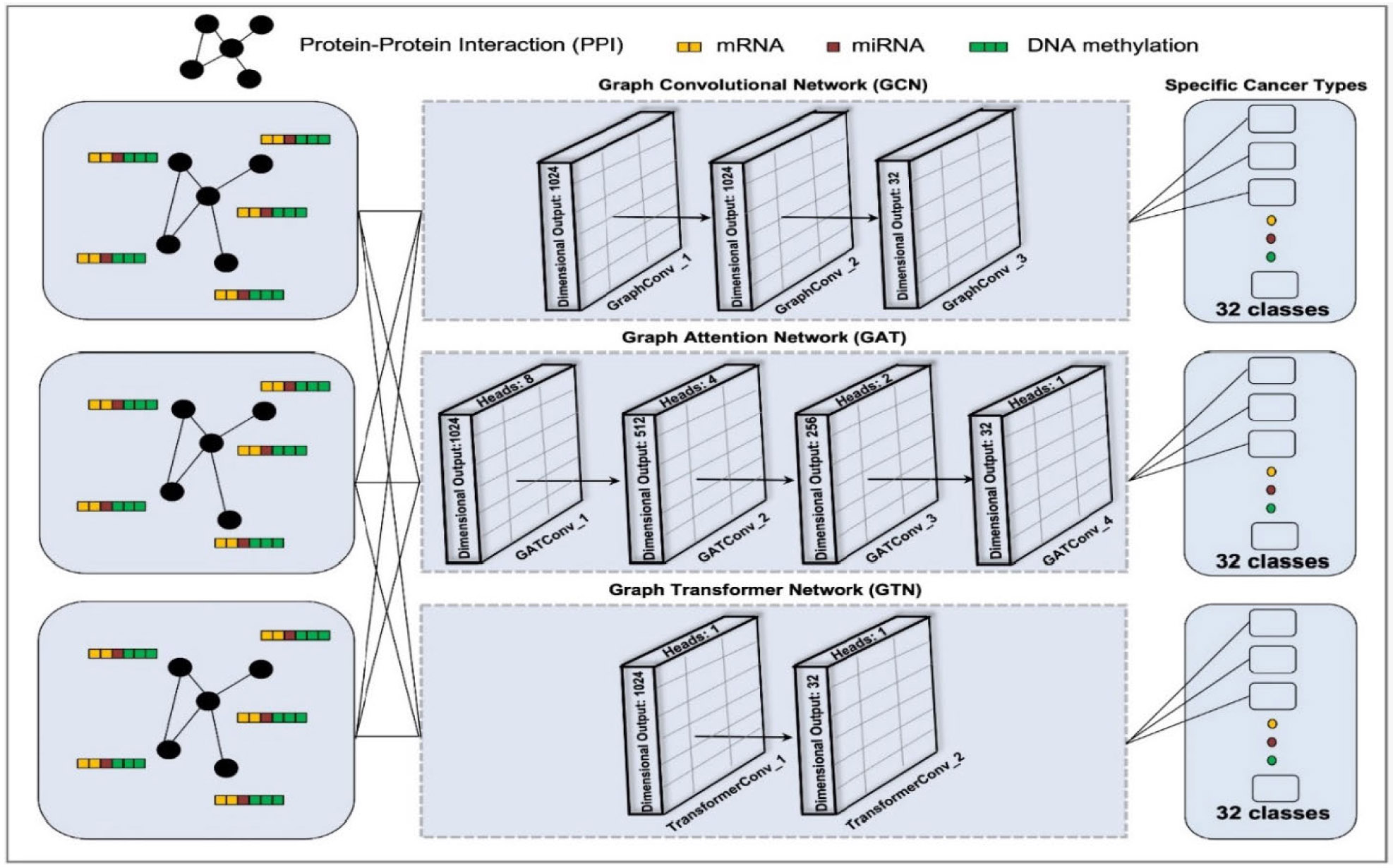
Architectures of the MOGCN, MOGAT, and MOGTN models for multiclass cancer classification.

The update rule for the transformer layer in GTNs is as follows [57]:

(6) $$H^{\left(l+1\right)}=\text{Layer}(H^{\left(l\right)},A)$$

where incorporating self-attention mechanisms [14] is given with:

(7) $$\text{Attention}(Q,K,V)=\text{softmax}\left(\frac{QK^{T}}{{\sqrt{d}}_{k}}\right)V$$

where Q, K , V are the query matrix, the key matrix, and the value matrix respectively, and $d_{k}$ denotes the dimension of the key vectors,

The architectures of MOGCN, MOGAT, and MOGTN are depicted in Figure 3.

## III. RESULTS

### A. EXPERIMENTAL SETUP

The GAT architecture consists of four GATConv layers, configured as follows. The first layer has 1024 output channels and 8 attention heads, followed by 512 channels and 4 attention heads in the second layer, 256 channels and 2 attention heads in the third layer, and the final layer has 32 channels with 1 attention head. Batch Normalization and Leaky ReLU activation are used after each layer, and dropout layers with 0.5 dropout rate are added. Adam optimizer with an initial learning rate of 0.001 is used, and a learning rate scheduler (ReduceLROnPlateau) is employed to adjust the learning rate dynamically based on the validation accuracy. The model was trained for 100 epochs. The hyperparameters were optimized based on the grid search approach.

The GCN model was designed with three GraphConv layers, each with 1024 hidden features and ReLU activation function. Dropout layers with a 0.5 dropout rate are applied after each layer. Adam optimizer with a learning rate of 0.001 is employed. The model was trained for 100 epochs.

The GTN architecture comprises two TransformerConv layers. The first TransformerConv layer outputs 1024 channels, and the second TransformerConv layer reduces the dimensionality to match the number of classes for final classification. Each TransformerConv layer is responsible for different portions of the input features and also collects data from adjacent nodes in the graph. Dropout with a 0.5 dropout rate is applied for each layer. We used Adam optimizer with a learning rate of 0.001, and ReduceLROnPlateau was used as a learning rate scheduler to adjust the learning rate based on the validation accuracy dynamically. We trained the model for 100 epochs.

All the models were coded in Python using the PyTorch Geometric library. The multi-omics dataset was split into training, validation, and test sets, and the performance of the models was assessed using a five-fold cross-validation approach.

### B. EXPERIMENTAL EVALUATION

The performance of the graph-based models for different combinations of multi-omics data when patients’ correlation matrices are used as a graph structure is presented in Table 2. The highest performance is achieved by LASSO-MOGAT for the combination of mRNA or RNA-Seq, miRNA, and DNA methylation. In this combination, LASSO-MOGAT achieved an accuracy of 95.90%. LASSO-MOGCN achieved comparable performance with 95.39% accuracy when using multi-omics data as input. Among the single omics data, LASSO-MOGAT achieved the highest accuracy of 94.88% when using DNA methylation alone. These results indicate that the combination of three omics data types is able to capture the most comprehensive biological signal. LASSO-MOGTN achieved good performance when combining mRNA or RNA-Seq with other omics data types, such as miRNA and DNA methylation. Therefore, integration of mRNA or RNA-Seq data might be more beneficial for cancer classification using LASSO-MOGTN with the correlation matrix-based approach. On the other hand, LASSO-MOGTN might fail to take full advantage of the synergistic potential of multiple omics data.

The low values of standard deviations for the experiments imply stable performance across different test sets in five-fold cross-validation. Generally, the models’ performance on the combination of multi-omics data is greater than on single omics data, where the lowest performance is achieved when the models are applied to miRNA data alone.

Table 3 presents the performance of the models when using the PPI network as a graph structure for classifying 31 types of cancer and normal tissues. LASSO-MOGCN achieved the best performance on the omics combination of mRNA or RNA-Seq and DNA methylation, with an accuracy of 95.35%. The performance is comparable to that achieved when the correlation matrices are used as a graph structure, presented in Table 2. For LASSO-MOGAT, even though single omics data types like DNA methylation or mRNA yield high accuracy, the results are significantly enhanced when all three omics data types are combined. This combination yields the best results with an accuracy of 95.74%. For LASSO-MOGTN, the highest performance is achieved when combining mRNA with DNA methylation. The integration of the three omics types yielded comparable, but slightly lower performance for LASSO-MOGTN.

In summary, LASSO-MOGCN model obtained the best performance when using the features combination of mRNA or RNA-Seq, miRNA, and DNA methylation based on the correlation matrix as a graph structure. LASSO-MOGTN based on the correlation matrix has a lower performance compared to LASSO-MOGCN. Because of its structure or the unique characteristics of the data, the GTN model may not have been able to fully utilize the potential of correlation matrices, and it is more suited to leveraging the interactions captured in PPI networks. LASSO-MOGAT achieved the best performance compared to the other models when using either the PPI network or the correlation matrix.

Table 4 presents a comparison of the performance of LASSO-MOGAT to related works in the literature for cancer classification based on different multi-omics data. The results indicate that the proposed LASSO-MOGAT model has the highest accuracy of 95.90% for 32 cancer classes using mRNA, miRNA, and DNA methylation data. The work by Mostavi et al. [58] based on Convolutional Neural Network models (1D-CNN, 2D-Vanilla-CNN, and 2D- Hybrid-CNN) achieved comparable but slightly lower accuracy for classification of 34 cancer types, where 1D-CNN and 2D-Hybrid-CNN models achieve 95.50% and 95.70%, respectively. The GCNN-PPI model proposed by Ramirez et al. [22] employs PPI networks as a graph structure and mRNA data and has lower performance in comparison to LASSO-MOGAT. Similarly, Kaczmarek et al. [33] proposed GTN for classification of 12 cancer types, achieving 93.56% accuracy.

A Wilcoxon signed rank test was adopted for statistical analysis of the experimental results, based on the distribution of the values for the performance metrics. The null hypothesis states that there is no statistically significant difference between the performance of a Graph Attention Network (GAT) model when applied to multi-omics data versus single-omics data. The results from the hypothesis testing for cases where a correlation matrix and PPI networks are used as a graph structure are presented in Table 5. The Wilcoxon test is performed across all performance metrics Accuracy, Precision, Recall, F1 score, ROC-AUC, and AUPR. The cells with asterisks in Table 5 indicate rejection of the null hypothesis at a *p*-value <0.05. The reported *p*-values and adjusted *p*-values indicate that the null hypotheses are rejected for all metrics, i.e., there is a statistically significant difference in the median values obtained by the multi-omics models in comparison to single-omics models.

## IV. CONCLUSION

This study presents a novel approach for integrating multi-omics data using graph-based neural network models for cancer classification. The approach employs Differential Gene Expression and LASSO regression to select the most significant features in multi-omics data, and leverages graph-based architectures GCN, GAT, and GTN to model biological interactions within multi-omics data. Experimental results demonstrate that the LASSO-MOGAT model based on integrating mRNA, miRNA, and DNA methylation data outperforms models using single omics data and achieves the highest accuracy of class separation. These results highlight the importance of multi-omics data integration for cancer classification, particularly when combined with correlation matrices structures in GAT models.

## Figures and Tables

**TABLE 1.** Pipeline for data processing.

Datatype	mRNA	miRNA	DNA Methylation
Original Features	60,660	1881	485,577
Differentially Expressed Analysis (DEGs)	26,768	-	-
LIMMA Model (Selected Features)	-	-	139,321
LASSO Regression Model (Selected Features)	520	-	393
All Tumor Samples and Normal Tissues	10,668	10,465	9,171
Unique Tumor Samples and Normal Tissues	10,667	10,465	8,674
Integrated Data	8,464 Samples and 2,794 Features		
Network Nodes and Edges	504 Nodes and 343 Edges		

**TABLE 2.** Performance of the proposed approaches based on correlation matrices (Mean ± Standard Deviation).

Data Types	Data Type	LASSO- MOGCN	LASSO- MOGAT	LASSO- MOGTN
Single Omics Data	mRNA or RNA-Seq	93.44±0.5	93.88±0.4	92.53±0.2
Single Omics Data	miRNA	87.92±0.4	89.59±0.7	84.78±0.6
Single Omics Data	DNA methylation	91.70±0.8	94.88±0.3	93.43±0.6
Multi-Omics Data	mRNA or RNA-Seq and miRNA	94.05±0.3	94.55±0.4	92.46±0.6
Multi-Omics Data	mRNA or RNA-Seq and DNA methylation	95.30±0.4	95.67±0.2	**95.01±0.2**
Multi-Omics Data	miRNA and DNA methylation	93.96±0.2	94.81±0.5	92.72±0.6
**Multi-Omics Data**	mRNA or RNA-Seq, miRNA and DNA methylation	**95.39±0.3**	**95.90±0.1**	94.06±0.7

**TABLE 3.** Performance of the proposed approaches based on PPI Network (Mean ± Standard Deviation).

Data Types	Data Type	LASSO- MOGCN	LASSO- MOGAT	LASSO- MOGTN
Single Omics Data	mRNA or RNA-Seq	93.36±0.2	93.67±0.4	93.37±0.5
Single Omics Data	miRNA	88.26±0.3	89.15±0.3	88.58±0.3
Single Omics Data	DNA methylation	94.23±0.2	94.61±0.3	94.38±0.1
Multi-Omics Data	mRNA or RNA-Seq and miRNA	93.98±0.1	94.11±0.7	93.97±0.3
Multi-Omics Data	mRNA or RNA-Seq and DNA methylation	**95.35±0.4**	95.53±0.2	**95.29±0.2**
Multi-Omics Data	miRNA and DNA methylation	93.72±0.6	94.80±0.7	94.37±0.5
**Multi-Omics Data**	mRNA or RNA-Seq, miRNA and DNA methylation	95.10±0.5	**95.74±0.3**	95.25±0.4

**TABLE 4.** Performance metrics for related Single-Omics and Multi-Omics graph methods.

Authors & Models	Classes	Multi-Omics Data Type			
		mRNA	miRNA	DNA methylation	Accuracy Mean ± std
**Proposed LASSO-MOGAT**	32 Classes	√	√	√	**95.90±0.1**
Mostavi et al.[58] 1D-CNN	34 Classes	√	-	-	95.50±0.1
2D-Vanilla-CNN		√	-	-	94.87±0.4
2D-Hybrid-CNN		√	-	-	95.70±1.0
Ramirez et al.[22] GCNN-PPI graph	34 Classes	√	-	-	88.98±0.9
GCNN-PPI±singleton graph		√	-	-	94.61±1.0
Kaczmarek et al. [33] GTN	12 Classes	√	√	-	93.56±0.9

**TABLE 5.** Statistical analysis of GAT models using Multi-Omics vs. Single-Omics data based on correlation matrix and PPI network.

	Performance metrics	*p*-value	Adjusted *p*-value
**GAT method using multi omics data vs GAT method using single omics data based on correlation matrices**	**Accuracy**	*3.892e-18	*3.896e-18
	**Precision**	*3.896e-18	*3.896e-18
	**Recall**	*3.896e-18	*3.896e-18
	**F1 score**	*3.896e-18	*3.896e-18
	**ROC-AUC**	*3.896e-18	*3.896e-18
	**AUPR**	*3.896e-18	*3.896e-18
**GAT method using multi omics data vs GAT method using single omics data based on PPI**	**Accuracy**	*3.873e-18	*1.168e-17
	**Precision**	*3.471e-16	*6.942e-16
	**Recall**	*8.802e-14	*1.056e-13
	**F1 score**	*1.419e-15	*2.128e-15
	**ROC-AUC**	*8.691e-11	*8.691e-11
	**AUPR**	*3.896e-18	*1.168e-17

**TABLE 6.** Performance metrics of the proposed LASSO-MOGCN approach based on correlation matrices.

Data Types	Data Type	Accuracy	Precision	Recall	F1 Score
Single Omics Data	mRNA or RNA-Seq	0.9344±0.005	0.9197±0.004	0.9119±0.007	0.9129 ± 0.003
Single Omics Data	miRNA	0.8792±0.004	0.8611±0.015	0.8500±0.015	0.8521 ± 0.015
Single Omics Data	DNA methylation	0.9170 ± 0.008	0.8838 ± 0.027	0.8663 ± 0.021	0.8657 ± 0.023
Multi-Omics Data	mRNA or RNA-Seq and miRNA	0.9405 ± 0.003	0.9331 ± 0.008	0.9204 ± 0.013	0.9224 ± 0.011
Multi-Omics Data	mRNA or RNA-Seq and DNA methylation	0.9530 ± 0.004	0.9392 ± 0.009	0.9309 ± 0.005	0.9320 ± 0.003
Multi-Omics Data	miRNA and DNA methylation	0.9396 ± 0.002	0.9255 ± 0.019	0.9109 ± 0.008	0.9147 ± 0.012
**Multi-Omics Data**	mRNA or RNA-Seq, miRNA and DNA methylation	**0.9539 ± 0.003**	**0.9462 ± 0.010**	**0.9374 ± 0.006**	**0.9380 ± 0.004**

**TABLE 7.** Performance metrics of the proposed LASSO-MOGCN approach based on PPI network.

Data Types	Data Type	Accuracy	Precision	Recall	F1 Score
Single Omics Data	mRNA or RNA-Seq	0.9336 ± 0.002	0.9175 ± 0.003	0.9049 ± 0.013	0.9059 ± 0.006
Single Omics Data	miRNA	0.8826 ± 0.003	0.8572 ± 0.015	0.8424 ± 0.007	0.8446 ± 0.009
Single Omics Data	DNA methylation	0.9423 ± 0.002	0.9205 ± 0.017	0.9116 ± 0.015	0.9129 ± 0.015
Multi-Omics Data	mRNA or RNA-Seq and miRNA	0.9398 ± 0.001	0.9275 ± 0.017	0.9197 ± 0.011	0.9201 ± 0.013
Multi-Omics Data	mRNA or RNA-Seq and DNA methylation	**0.9535 ± 0.004**	**0.9445 ± 0.011**	**0.9291 ± 0.009**	**0.9328 ± 0.011**
Multi-Omics Data	miRNA and DNA methylation	0.9372 ± 0.006	0.9170 ± 0.019	0.9024 ± 0.018	0.9058 ± 0.018
**Multi-Omics Data**	mRNA or RNA-Seq, miRNA and DNA methylation	0.9510 ± 0.005	0.9348 ± 0.012	0.9227 ± 0.011	0.9250 ± 0.011

**TABLE 8.** Performance metrics of the proposed LASSO-MOGAT approach based on correlation matrices.

Data Types	Data Type	Accuracy	Precision	Recall	F1 Score
Single Omics Data	mRNA or RNA-Seq	0.9388 ± 0.004	0.9169 ± 0.006	0.9283 ± 0.013	0.9205 ± 0.006
Single Omics Data	miRNA	0.8959 ± 0.007	0.8665 ± 0.009	0.8636 ± 0.011	0. 8613 ± 0.006
Single Omics Data	DNA methylation	0.9488 ± 0.003	0.9226±0.011	0.9294 ± 0.013	0.9242 ± 0.011
Multi-Omics Data	mRNA or RNA-Seq and miRNA	0.9455 ± 0.004	0.9229 ± 0.014	0.9258 ± 0.008	0.9224 ± 0.011
Multi-Omics Data	mRNA or RNA-Seq and DNA methylation	0.9567 ± 0.002	0.9316 ± 0.012	0.9375 ± 0.017	0.9331 ± 0.015
Multi-Omics Data	miRNA and DNA methylation	0.9481 ± 0.005	0.9202 ± 0.010	0.9248 ± 0.007	0.9189 ± 0.009
**Multi-Omics Data**	mRNA or RNA-Seq, miRNA and DNA methylation	**0.9590 ± 0.001**	**0.9438 ± 0.008**	**0.9445 ± 0.009**	**0.9420 ± 0.008**

**TABLE 9.** Performance metrics of the proposed LASSO-MOGAT approach based on PPI Network.

Data Types	Data Type	Accuracy	Precision	Recall	F1 Score
Single Omics Data	mRNA or RNA-Seq	0.9367 ± 0.004	0.9175 ± 0.007	0.9185 ± 0.013	0.9149 ± 0.010
Single Omics Data	miRNA	0.8915 ± 0.003	0.8512 ± 0.007	0.8513 ± 0.007	0.8482 ± 0.007
Single Omics Data	DNA methylation	0.9461 ± 0.003	0.9185 ± 0.016	0.9188 ± 0.017	0.9170 ± 0.016
Multi-Omics Data	mRNA or RNA-Seq and miRNA	0.9411 ± 0.007	0.9160 ± 0.023	0.9158 ± 0.014	0.9128 ± 0.015
Multi-Omics Data	mRNA or RNA-Seq and DNA methylation	0.9553 ± 0.002	0.9395 ± 0.004	0.9428 ± 0.007	0.9399 ± 0.006
Multi-Omics Data	miRNA and DNA methylation	0.9480 ± 0.007	0.9134 ± 0.023	0.9154 ± 0.019	0.9126 ± 0.021
**Multi-Omics Data**	mRNA or RNA-Seq, miRNA and DNA methylation	**0.9574 ± 0.003**	**0.9450 ± 0.009**	**0.9354 ± 0.011**	**0.9362 ± 0.011**

## APPENDIX A

### A. DATA COLLECTION

For collecting the multi-omics from the Pan-Cancer Atlas we used the GDC query tool from the TCGA biolinks library. The GDC query function requires the following parameters: project, legacy, data.category, data.type, and sample.type. The project parameter was set to “TCGA-∗”, encompassing all 33 TCGA projects (cancer types in addition to normal tissues). The legacy parameter was set to True, indicating that the query should retrieve unaltered data from the TCGA data repository. The data.category parameter included transcriptome profiling for mRNA or RNA-Seq, miRNA datasets, and DNA Methylation category. The data.type argument was set to Gene Expression Quantification for mRNA or RNA-Seq data, miRNA Expression Quantification for miRNA data, and Methylation Beta Value for DNA Methylation data. The sample.type parameter was set to “Primary Solid Tumor” and “Solid Tissue Normal” to extract omics data from both tumor and normal samples.

**TABLE 10.** Performance metrics of the proposed LASSO-MOGTN approach based on correlation matrices.

Data Types	Data Type	Accuracy	Precision	Recall	F1 Score
Single Omics Data	mRNA or RNA-Seq	0.9253 ± 0.002	0.9051 ± 0.013	0.9014 ± 0.012	0.9007 ± 0.012
Single Omics Data	miRNA	0.8478 ± 0.006	0.8267 ± 0.021	0.8018 ± 0.009	0.8088 ± 0.015
Single Omics Data	DNA methylation	0.9343 ± 0.006	0.9178 ± 0.019	0.9017 ± 0.022	0.9035 ± 0.021
Multi-Omics Data	mRNA or RNA-Seq and miRNA	0.9246 ± 0.006	0.9116 ± 0.018	0.8989 ± 0.015	0.9027 ± 0.017
Multi-Omics Data	mRNA or RNA-Seq and DNA methylation	**0.9501 ± 0.002**	**0.9419 ± 0.010**	**0.9319 ± 0.009**	**0.9347 ± 0.009**
Multi-Omics Data	miRNA and DNA methylation	0.9272 ± 0.006	0.9152 ± 0.010	0.8991 ± 0.011	0.9044 ± 0.009
**Multi-Omics Data**	mRNA or RNA-Seq, miRNA and DNA methylation	0.9406 ± 0.007	0.9204 ± 0.026	0.9089 ± 0.024	0.9101 ± 0.023

**TABLE 11.** Performance metrics of the proposed LASSO-MOGTN approach based on PPI Network.

Data Types	Data Type	Accuracy	Precision	Recall	F1 Score
Single Omics Data	mRNA or RNA-Seq	0.9337 ± 0.005	0.9153 ± 0.006	0.9040 ± 0.013	0.9043 ± 0.010
Single Omics Data	miRNA	0.8858 ± 0.003	0.8666 ± 0.008	0.8519 ± 0.012	0.8560 ± 0.009
Single Omics Data	DNA methylation	0.9438 ± 0.001	0.9253 ± 0.014	0.9151 ± 0.008	0.9182 ± 0.010
Multi-Omics Data	mRNA or RNA-Seq and miRNA	0.9397 ± 0.003	0.9338 ± 0.009	0.9225 ± 0.010	0.9231 ± 0.011
Multi-Omics Data	mRNA or RNA-Seq and DNA methylation	**0.9529 ± 0.002**	**0.9412 ± 0.005**	**0.9351 ± 0.005**	**0.9361 ± 0.005**
Multi-Omics Data	miRNA and DNA methylation	0.9437 ± 0.005	0.9323 ± 0.021	0.9177 ± 0.016	0.9210 ± 0.018
**Multi-Omics Data**	mRNA or RNA-Seq, miRNA and DNA methylation	0.9525 ± 0.004	0.9386 ± 0.020	0.9302 ± 0.015	0.9318 ± 0.018

### B. EXPERIMENTAL RESULTS

This section presents a more comprehensive comparative analysis of the performance of LASSO-MOGCN, LASSO-MOGAT, and LASSO-MOGTN based on a set of performance metrics, including precision, recall, and F1 score, in addition to the results based solely on accuracy presented in Section IV. Tables 6 and 7 contain the values of the performance metrics for LASSO-MOGCN based on using patients’ correlation matrices and PPI networks as graph structures, respectively. By analogy, Tables 8 and 9 present the performance metrics for LASSO-MOGAT, and Tables 10 and 11 present the performance metrics for LASSO-MOGTN, respectively.
